# Dataset development of pre-formulation tests on fast disintegrating tablets (FDT): data aggregation

**DOI:** 10.1186/s13104-023-06416-w

**Published:** 2023-07-03

**Authors:** Mehri Momeni, Saleh Rakhshani, Mohammadreza Abbaspour, Faezeh Alizadeh, Nafiseh Sheikhi, Faezeh GhorbanZadeh, Zahra Habibi, Hamed Tabesh

**Affiliations:** 1 Department of Medical Informatics, Faculty of Medicine, Mashhad University of Medical Sciences, Mashhad, Iran; 2 Department of pharmaceutics, school of pharmacy, Mashhad University of Medical Sciences, Mashhad, Iran; 3 Student research committee, Mashhad University of Medical Sciences, Mashhad, Iran

**Keywords:** Dataset development, Fast disintegrating tablets, Predictive model, Data extraction, Pre-formulation, Direct compression

## Abstract

**Objectives:**

Tablet manufacturing development is costly, laborious, and time-consuming. Technologies related to artificial intelligence like ,predictive model ,can be used in the control process to facilitate and accelerate the tablet manufacturing process. predictive models have become popular recently. However, predictive models need a comprehensive dataset of related data in the field, due to the lack of a dataset of tablet formulations, the aim of this study is to aggregate and integrate fast disintegration tablet’s formulation into a comprehensive dataset.

**Data description:**

The search strategy has been prepared between the years of 2010 to 2020, consisting of the keyword’s ‘formulation’ ,‘disintegrating’ and ‘Tablet’, as well as their synonyms. By searching four databases, 1503 articles were retrieved, from these articles only 232 articles met all of the study’s criteria. By reviewing 232 articles, 1982 formulations have been extracted, afterward pre-processing and cleaning data, contain steps of unifying the name and units, removing inappropriate formulations by an expert, and finally, data tidying was done on data. The developed dataset contains valuable information from various FDT’s formulations, which can be used in pharmaceutical studies that are critical to the discovery and development of new drugs. this method can be applied to aggregate datasets from the other dosage forms.

## Objective

The pharmaceutical industry, as one of the largest industries in the world, seeks on one hand to discover and develop new drugs, and on the other hand, to research and improve existing drug formulations with optimal methods that meet the requirements of treatment and disease. Therefore, simplifying and streamlining the pre-formulation process has become essential and important for pharmaceutical experts in this industry [1, 2].

Among the most popular solid dosage forms, including capsules and tablets, tablets are the most frequently used due to their ease of swallowing [3]. Another significant advantage of tablets is their flexibility in addressing various disease conditions. Changes in the composition of excipients lead to the production of different tablets with different functions. For example, immediate-release tablets or modified-release tablets can be created by altering the excipients. According to the United States Pharmacopeia (USP) definition, immediate-release tablets are a type of tablets that, when administered and placed near gastrointestinal fluids, disintegrate and release their ingredients in less than 3 min. The disintegration time test is sufficient to evaluate this type of tablet formulation [4]. The development of this kind of tablet involves pre-formulation studies through trial and error, which are expensive, time-consuming, and laborious. Moreover, these current methods are known to be a source of environmental pollution. Executing these experiments has become a major challenge for the pharmaceutical industry [1, 2].

In the last decade, there has been a growing use of appropriate techniques that employ machine learning algorithms to predict formulations in research. Machine learning techniques are superior to conventional statistical methods as they are learnable and can automate processes, leading to improved development speed, optimized formulation, and significant cost savings [5]. One such technique gaining considerable attention recently is deep learning, which is a subfield of machine learning that trains artificial neural networks to automatically learn and make complex predictions or decisions from data. Studies conducted over the years have demonstrated that these algorithms yield better results compared to other machine learning methods in predicting the disintegration or dissolution time of tablets, drug solubility in water, and the detection of new medicines [6–11].

As an example, in study [11], regression models were used to predict the correct drug formulation. The study introduced a deep neural network trained on two types of drug forms: oral fast disintegrating films (OFDF) and oral sustained release matrix tablets (SRMT). Additionally, the deep learning method was compared to six other machine learning algorithms.

In study [8], deep learning methods (DNN) and artificial neural networks (ANN) were employed to design a quantitative model for predicting the disintegration time of oral fast disintegrating tablets using the Direct Compression method.

In study [9], a recurrent neural network was utilized to predict molecular properties by examining the solubility of the drug in water based on its molecular structure.

The initial step in developing a prediction model involves data collection. In this particular case, due to the limited availability of a gathered dataset, our study aimed to create a dataset by aggregating information from articles on fast-disintegrating tablets (FDT) formulations. We believe that this effort is necessary to meet the pharmaceutical industry’s needs for automating medicinal processes, which require the utilization of machine learning techniques, including deep learning, to predict the disintegration time of FDT, an important specification in pre-formulation studies. Given the requirement for a comprehensive dataset, the primary objective of this study was to compile data and create a dataset consisting of FDT formulations and their corresponding properties based on previous studies.

## Data description

Given the extensive nature of the pharmaceutical technologies field and the absence of a comprehensive dataset encompassing pharmaceutical formulations and their corresponding control test values, which is a key requirement for developing predictive models, we performed a systematic search across four databases. Additionally, the selection of tablet pharmaceutical form was based on its widespread usage, and within the tablet category, fast-disintegrating tablets were chosen. The evaluation of these tablets focused on their disintegration time, fragility, and hardness, which are considered crucial parameters.

**Fig. 1:**
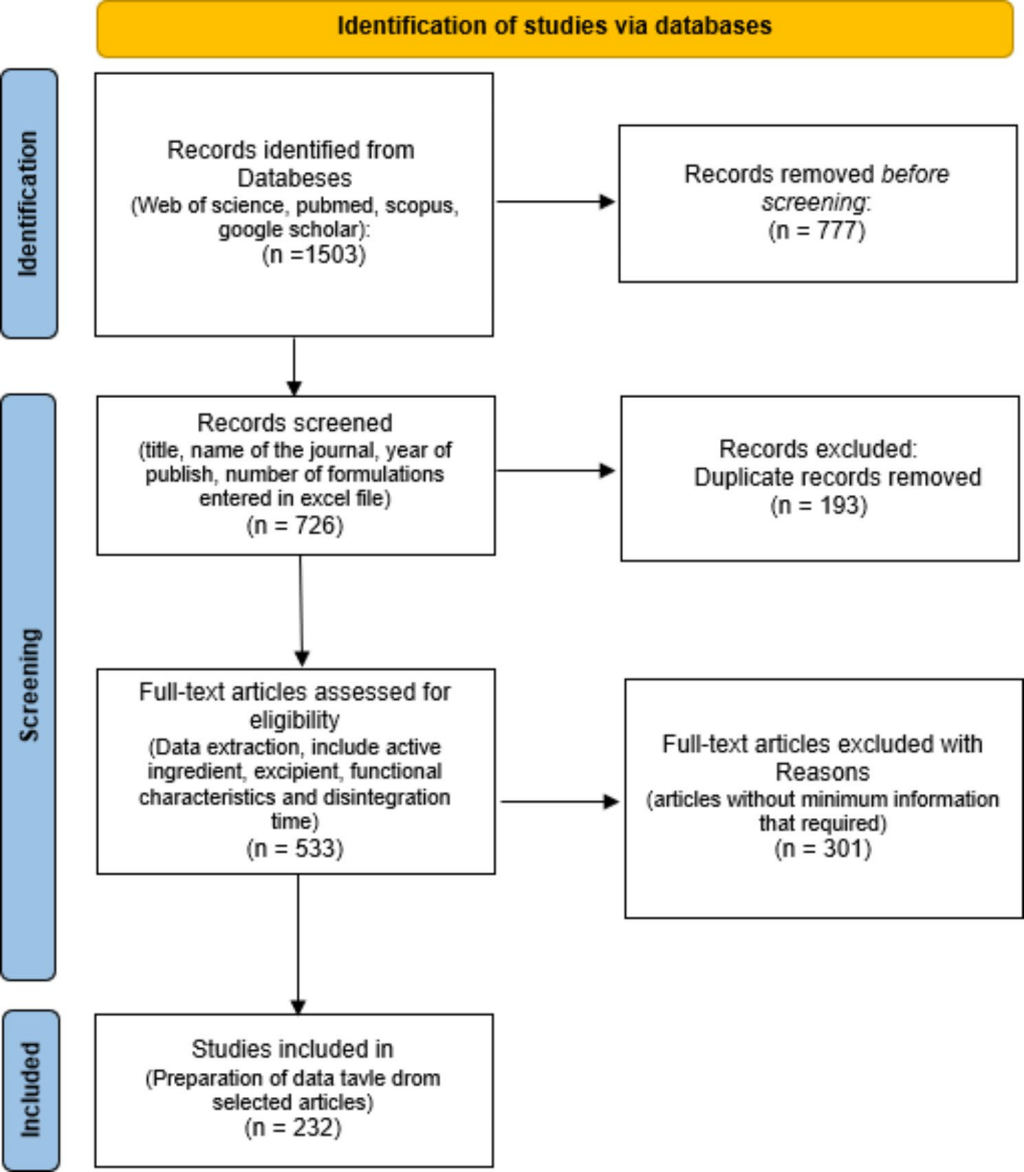
Steps of study implementation

A total of 1,503 articles were retrieved through the database search. During the initial review, which involved a thorough examination of the articles’ full texts to identify those that analyzed formulations with the desired structural values and characteristics, 726 articles were identified. Among these, 193 articles were found to be duplicated across multiple databases. Subsequently, 523 articles proceeded to the next step for a detailed assessment of their full texts, specifically focusing on the inclusion criteria for adding formulations to the dataset. As a result, 301 articles did not meet all the inclusion criteria and were subsequently excluded from the study. The summarized steps can be visualized in Fig. 1.

After reviewing 232 articles, a total of 1,982 formulations were extracted. An overview of the dataset is provided in Table 1. The formulation information, including the name and content of Active Pharmaceutical Ingredients (API), as well as other excipients, process details, and quality control properties, were recorded in the dataset. Each formulation in the final dataset contains the following features: API name, Dose, Amount of Excipients (each excipient as a separate column), Total Weight, Hardness, Friability, Thickness, Wetting Time, Drug Content, Disintegration Time, Content Uniformity, Water Absorption Ratio, Mixing Time, Diameter, Bulk Density, Tapped Density, Carr’s Compressibility Index, Hausner Ratio, Angle Of Repose, Tablet Porosity, Assay, Moisture Content, Dispersion Time, and Cumulative Drug Release.

Currently, tablet manufacturing processes for achieving the optimal formulation traditionally involve multiple trials and errors, as indicated by existing research. However, the utilization of deep learning techniques as part of the Quality by Design (QBD) principles in the pharmaceutical industry necessitates a comprehensive database of relevant formulations, which was previously unavailable. In this study, we have created a dataset by aggregating data to enable advanced analytics concerning the presentation of the optimal formulation. To the best of our knowledge, this is the first instance of such an endeavour.

The dataset contains valuable information regarding various formulations of fast-disintegrating tablets, which can be utilized in other studies. Furthermore, the dataset can be used to conduct an optimal analysis of formulation steps. The methodology employed in this study can also be applied to develop datasets for other dosage forms, serving as a prerequisite and introduction to further research in the field of modelling drug formulations.

In future work, this dataset will be employed to construct a prediction model using machine learning and deep learning techniques to forecast the disintegration time of fast-disintegrating tablets.

**Fig. 2:**
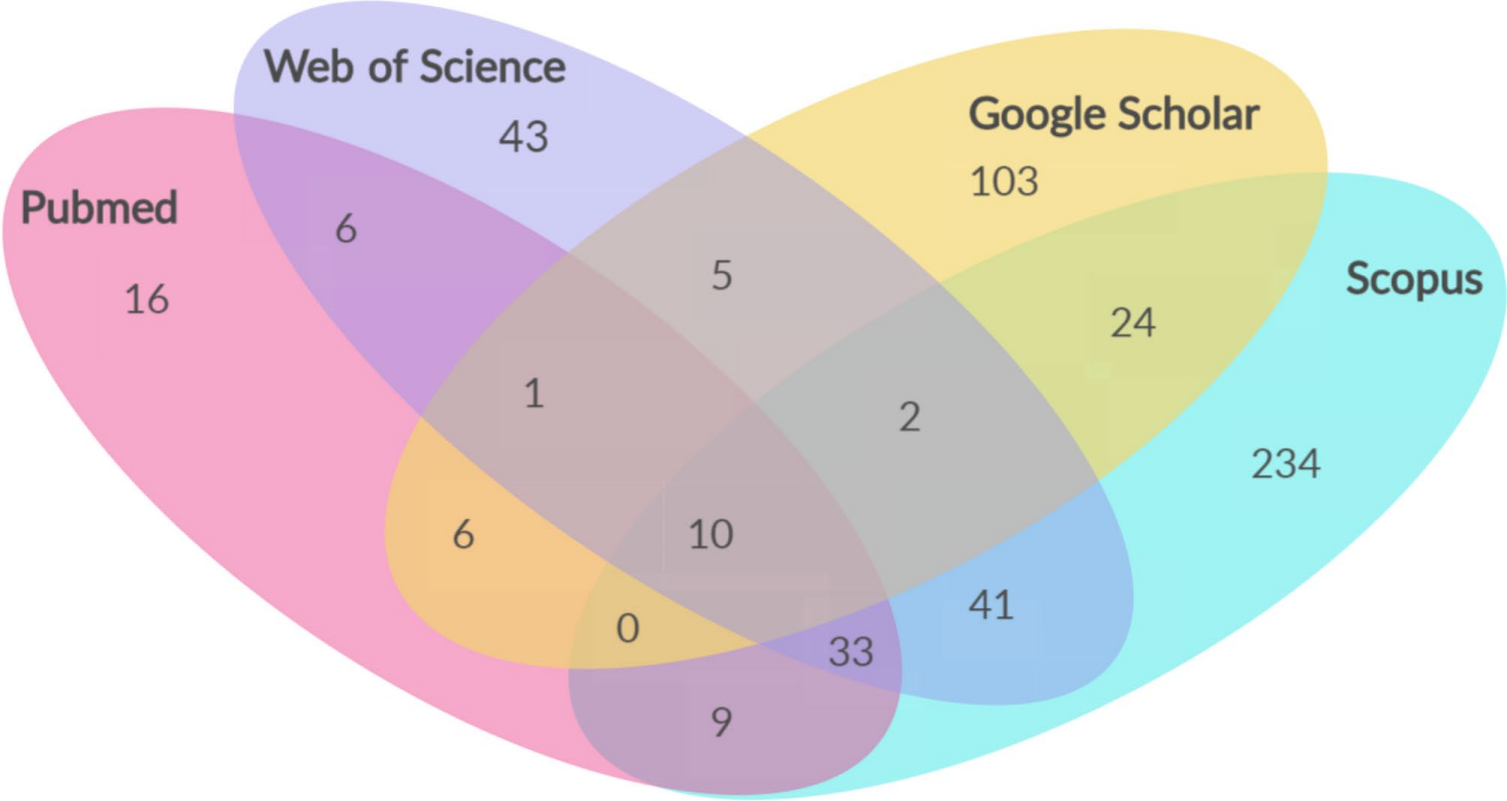
Number of articles extracted from each database

Another notable finding from our study, as depicted in Fig. 2, is that a significant proportion of articles were found in the Scopus and Google Scholar databases. By conducting searches specifically in these databases, we were able to access the majority of the articles included in our study. This highlights the importance of utilizing these databases as valuable sources of research literature.

**Table 1:** Overview of data files/data sets

Label	Name of data file/data set	File types (file extension)	Data repository and identifier (DOI or accession number)
*Data file 1*	*final Data All Exipients.tab*	*MS Excel file (.xlsx)*	Harvard Dataverse (10.7910/DVN/TUSJYB) (12)
*Data file 2*	*Method.docx*	*MS word file (.docx)*	Harvard Dataverse (10.7910/DVN/TUSJYB)(12)
*Data file 3*	Figure 1 steps of study implementation	*Picture files (.png)*	Harvard Dataverse (10.7910/DVN/TUSJYB)(12)
*Data file 4*	*DataArticlmanuscriptRef.enl*	*Endnote Ref file (.enl)*	Harvard Dataverse (10.7910/DVN/TUSJYB)(12)

**Limitations **In selecting the formulations of the articles, there were limitations that led to the exclusion of some formulations or even the entire article in data extraction. • Some articles did not report the main features of interest that were mentioned as inclusion criteria for this article. • A large number of articles did not use direct compression as the method for material blending. • Some articles reported the response variables as dispersion time instead of disintegration time, and as a result, these formulations were also excluded due to the different nature of these two response variables. 

## Data Availability

The data described in this Data note can be freely and openly accessed on **pre-formulation tests on fast disintegrating tablets (FDT)** under (10.7910/DVN/TUSJYB). Please see Table 1 and references [12] for details and links to the data.

## Abbreviations

FDT: Fast disintegrating tablets
USP: United States Pharmacopeia
API: Active Pharmaceutical Ingredient
QBD: Quality by Design
